# Surface Carbon Shell-Functionalized ZrO_2_ as Nanofiller in Polymer Gel Electrolyte-Based Dye-Sensitized Solar Cells

**DOI:** 10.3390/nano9101418

**Published:** 2019-10-04

**Authors:** Seung Man Lim, Juyoung Moon, Gyo Hun Choi, Uoon Chul Baek, Jeong Min Lim, Jung Tae Park, Jong Hak Kim

**Affiliations:** 1 Department of Chemical Engineering, Konkuk University, 120 Neungdong-ro, Gwangjin-gu, Seoul 05029, Korea; smlim01km@gmail.com (S.M.L.); jymoon01kd@gmail.com (J.M.); khchoi02km@gmail.com (G.H.C.); ucbaek04km@gmail.com (U.C.B.); jmlim03km@gmail.com (J.M.L.); 2 Department of Chemical and Biomolecular Engineering, Yonsei University, 50 Yonsei-ro, Seodaemun-gu, Seoul 03722, Korea

**Keywords:** atom transfer radical polymerization (ATRP), zirconium dioxide (ZrO_2_), carbon, polymer gel electrolyte (PGE), nanofiller (NF), dye-sensitized solar cell (DSSC)

## Abstract

We prepare dye-sensitized solar cells (DSSCs) fabricated with a poly (ethylene glycol) based polymer gel electrolytes (PGEs) incorporating surface carbon shell-functionalized ZrO_2_ nanoparticles (ZrO_2_-C) as nanofillers (NFs). ZrO_2_ are polymerized *via* atom transfer radical polymerization (ATRP) using poly (ethylene glycol) methyl ether methacrylate (POEM) as a scaffold to prepare the ZrO_2_-C through carbonization. The power conversion efficiency of DSSC with 12 wt% ZrO_2_-C/PGEs is 5.6%, exceeding that with PGEs (4.4%). The enhanced efficiency is attributed to Lewis acid-base interactions of ZrO_2_-C and poly (ethylene glycol), catalytic effect of the carbon shells of ZrO_2_-C, which results in reduced crystallinity, enhanced ion conductivity of electrolytes, decreased counterelectrode/electrolyte interfacial resistance, and improved charge transfer rate. These results demonstrate that ZrO_2_-C introduction to PGEs effectively improves the performance of DSSCs.

## 1. Introduction

Polymer gel electrolytes (PGEs), which are quasi-solid-state electrolytes, have promising properties such as appropriate ion conductivity, guaranteed stability, mechanical strength, and good electrode affinity [1,2,3,4]. These beneficial characteristics allow widespread PGEs application in electrochemical systems such as lithium-ion batteries, supercapacitors, and dye-sensitized solar cells (DSSCs) [5,6,7,8,9]. Despite the attractive electrochemical properties of PGEs, crystalline polymer matrices can limit ion diffusion in PGEs. Hindered charge transport causes decrease in ion conductivity and energy conversion efficiency [10,11].

A facile and effective way to improve the conductivity of PGEs is the introduction of nanomaterials to PGEs, such as inorganic metal oxides or carbonaceous materials, as nanofillers (NFs) [12,13,14,15,16]. With metal oxide NFs, the interaction between the Lewis acidic surfaces of metal oxides and Lewis basic polymers in PGEs reduces polymer self-interaction and thus prevents polymer crystallization; this increases the free volume for ion transport and hence enhances ion conductivity. Earth-abundant and cheap conductive carbonaceous materials have some merits compared to other nanomaterials as NFs because of their mechanical and electrochemical properties. Because these conductive carbon compounds create continuous conducting paths in the electrolyte by the overlapping of individual particles, high charge transfer rates arise. Furthermore, the catalytic activity of carbon compounds toward electrochemical reactions decreases the interfacial resistance and thereby improves the energy conversion efficiency [17,18,19].

Low-cost and high-performance DSSCs were reported by Grätzel and O’Regan in 1991, using mesoporous TiO_2_-deposited photoanodes, Pt-coated counter electrodes, and an iodine (I^−^/I_3_^−^) redox couple-based liquid electrolyte [20]. Despite their excellent photovoltaic performance, the poor long-term stability induced by solvent leakage and evaporation has restricted the commercialization of DSSCs. The replacement of liquid electrolytes with PGEs in DSSCs may solve these issues.

The functionalization of nanomaterials has received much attention because it easily alters nanomaterial properties. The surface polymerization of metal oxides *via* atom transfer radical polymerization (ATRP) with hydrophilic polymers can improve NFs particle dispersion in solvents or polymer matrices [21,22,23]. Moreover, these surface-polymerized nanomaterials are good candidates for obtaining carbonaceous–inorganic hybrid materials. Because of the advantageous characteristics of the ZrO_2_, such as their large band gap (~5–5.4 eV) and zero visible-light absorption, good physical and chemical durability, and reliable thermal stability, ZrO_2_ are good candidates for NFs [24,25,26].

In this work, we introduce carbon shell-functionalized ZrO_2_ (ZrO_2_-C) NFs to a PGEs and investigate the photovoltaic performances of ZrO_2_-C/PGE-based DSSCs. The surface-polymerized ZrO_2_ with poly (ethylene glycol) methyl ether methacrylate *via* ATRP is used as a scaffold to obtain the ZrO_2_-C through carbonization. Detailed properties of ZrO_2_-C are systematically characterized using transmission electron microscopy (TEM), Fourier-transform infrared (FTIR) spectroscopy, and thermogravimetric analysis (TGA). The DSSCs employing ZrO_2_-C/PGE electrolytes are fabricated and characterized in detail *via* electrochemical impedance spectroscopy (EIS), intensity modulated voltage spectroscopy (IMVS), incident photon-to-current efficiency (IPCE), and current-voltage curves, respectively.

## 2. Experimental Section

### 2.1. Materials

ZrO_2_ powder, 4-(dimethylamino) pyridine (DMAP, > 99%), 2-chloropropionyl chloride (CPC, > 97%), triethylamine (TEA, > 99.5%), methylene dichloride (MC, > 99.8%), copper (I) chloride (CuCl), 1,1,4,7,10,10-hexamethyl triethylenetetramine (HMTETA), dimethyl sulfoxide (DMSO), poly(ethylene glycol) methyl ether methacrylate (POEM, M_n_ = 500 g/mol), poly(ethylene glycol) (PEG, M_n_ = 100,000 g/mol), LiI, I_2_, 1-methyl-3-propylimidazolium iodide (MPII), H_2_PtCl_6_, and titanium diisopropoxide bis (acetylacetonate) were purchased from Sigma-Aldrich. Commercial TiO_2_ paste (Ti-Nanoxide, D20) and ruthenium dye (535-bisTBA, N719) were purchased from Solaronix, Switzerland. Ethanol, acetonitrile, chloroform, and 2-propanol were purchased from J.T. Baker. Deionized water (> 18 MΩm) was obtained with a water purification system made by Millipore Corporation. Fluorine-doped tin oxide (FTO, TEC-7) conducting substrates were purchased from Pilkington, France. All chemicals were used without further purification.

### 2.2. Surface Modification From ZrO_2_ to ZrO_2_-Cl

The surface hydroxyl groups (−OH) of ZrO_2_ were converted into chlorine groups (−Cl). First, 3.68 g of DMAP in 20 mL of MC was mixed with 2.8 mL of TEA in a round-bottom flask at 0 °C. Then, 10 g of ZrO_2_ in 50 mL of MC was added slowly to the DMAP solution, followed by N_2_ gas purging for 30 min. The mixed solution was reacted at room temperature for 24 h by stirring. After the reaction, the resulting solution was washed with methanol and centrifuged three to five times to remove impurities. Finally, the pink-colored ZrO_2_-Cl were obtained and dried in a vacuum oven overnight at room temperature.

### 2.3. Synthesis of ZrO_2_-g-POEM via ATRP

The surface-modified ZrO_2_-Cl were polymerized by POEM *via* ATRP. First, 20 mL of POEM in 10 mL of DMSO was prepared in a 100 mL round flask. Then, 0.0264 g of CuCl and 0.072 mL of HMTETA were added into the POEM solution, followed by 1 g of ZrO_2_-Cl; the mixture was then N_2_ gas-purged for 30 min. The mixture was then reacted in an oil bath at 90 °C for 24 h. The resulting solution was washed with methanol and centrifuged three to five times. Finally, the as-obtained ZrO_2_-*g*-POEM were dried in a vacuum oven at room temperature for 24 h.

### 2.4. Preparation of ZrO_2_-C

The ZrO_2_-*g*-POEM were used as a template to prepare ZrO_2_-C through carbonization. The 0.1 g of ZrO_2_-*g*-POEM were placed in a tube furnace and carbonized at 500 °C for 30 min in Ar flow with the heating rate of 3 °C min^−1^.

### 2.5. Preparation of ZrO_2_-C/PGEs

ZrO_2_-C/PGEs were prepared by solution-casting method. The ZrO_2_-C, PEG, LiI, MPII and I_2_ were dissolved in acetonitrile and cast on the Teflon-coated dish. The solvent was completely removed by vacuum drying. The concentrations of ZrO_2_-C were fixed at 9, 12 or 15 wt% relative to poly (ethylene glycol). The mole ratio of ether oxygen to iodine salt was fixed at 20, and the iodine content was fixed at 10 wt% with respect to the salt. As a control, 12 wt% ZrO_2_-*g*-POEM/PGEs and 12 wt% ZrO_2_/PGEs were prepared following the same procedures.

### 2.6. Fabrication of ZrO_2_-C/PGE-Based DSSCs

The photoanodes and counter electrodes were prepared as follow [27,28,29]. First, FTO glass substrates were rinsed with chloroform and 2-propanol for 30 min each. As a photoanode, 2 wt% titanium diisopropoxide bis(acetylacetonate) in butanol solution was spin-coated (1500 rpm, 30 s) onto the conductive side of the FTO glass, followed by calcination at 450 °C for 30 min. Commercial TiO_2_ paste was deposited onto the as-prepared photoanode *via* the doctor blade technique and dried at 50 °C and 80 °C for 1 h each. Subsequently, this photoanode was sintered at 450 °C for 30 min. Then, the as-obtained photoanode was immersed in 0.1 M N719 dye solution in ethanol at 50 °C for 3 h under dark condition. To prepare the counter electrode, 1 wt% H_2_PtCl_6_ in 2-propanol solution was spin-coated (1500 rpm, 30 s) onto the conductive side of the FTO glass followed by calcination at 450 °C for 30 min. Then, as prepared ZrO_2_-C/PGEs were drop casted on the photoanodes. The ZrO_2_-C/PGE-cast photoanode was superimposed on the counter electrode. Finally, the assembled cell was dried in a vacuum oven for a day at room temperature and sealed with an epoxy resin.

### 2.7. Characterization

The surface morphologies of the ZrO_2_-C were observed by transmission electron microscopy (TEM) (JEM-F200, JEOL, Tokyo, Japan). Thermogravimetric analysis (TGA) was performed by a TG209 F3 (NETZSCH) instrument. Fourier-transform infrared (FT-IR) spectra of the samples were collected using a PerkinElmer instrument from 4000 to 750 cm^−1^. Current density–voltage (*J-V*) measurements were acquired using a potentiostat (Compactstat.h, Ivium Technologies) equipped with a 150-W Xe lamp (Sunlite, ABET Technologies) as a light source. Electrochemical impedance spectroscopy (EIS) was measured at the open-circuit voltage (*V_oc_*) in the frequency range 0.01 Hz–100 kHz with an amplitude of 10 mV. Incident photo-to-electron conversion efficiency (IPCE) spectra were obtained by using a CE system (PEIPCE100S, HS-Technologies). Intensity-modulated voltage spectroscopy (IMVS) was used to check the charge recombination properties. The photovoltaic parameters were calculated by the following equations:

*FF = V_max_·J_max_/V_oc_·J_sc_* (1)

*η = V_max_·J_max_/Pin·100 = V_oc_·J_sc_·FF/P_in_·100* (2)

where *η* is the power conversion efficiency (%), *J*_sc_ is the short-circuit current density, *V*_oc_ is the open-circuit voltage, *P*_in_ is the incident light power, and *FF* is the fill factor. *J*_max_ and *V*_max_ are the current density and voltage in the *J-V* curves, respectively, at the maximum power output.

## 3. Results and Discussion

**Scheme 1:**
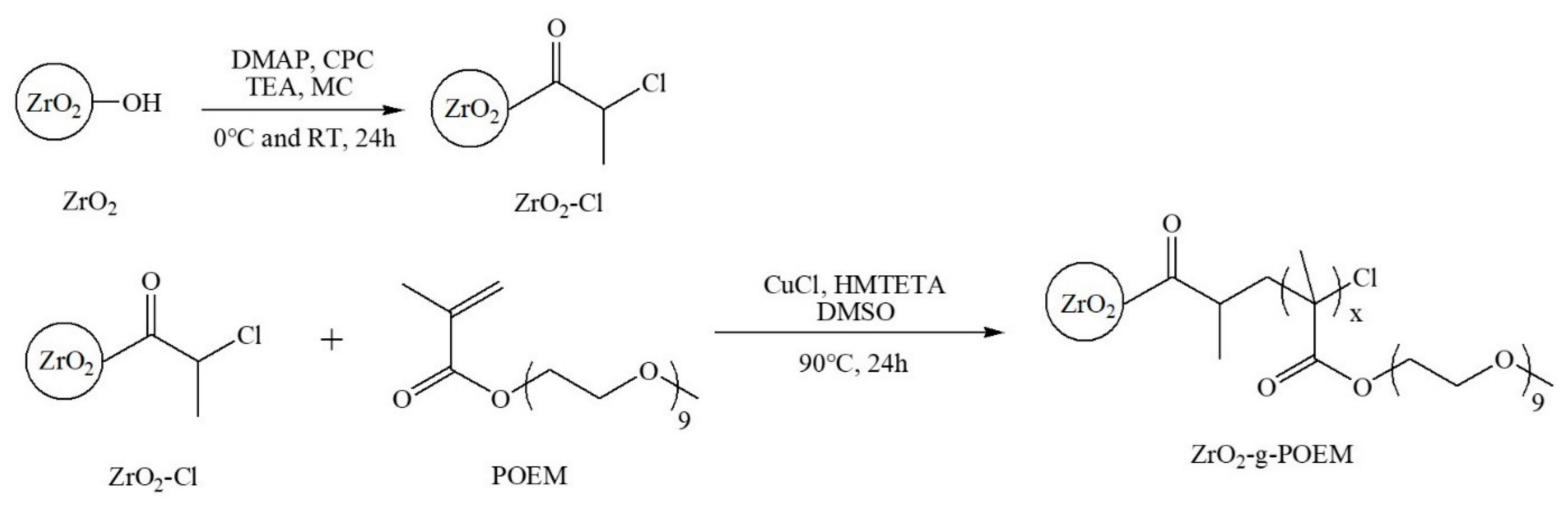
The reaction scheme for the graft polymerization of POEM from ZrO_2_ *via* ATRP.

Scheme 1 shows the reaction scheme by which the ZrO_2_ are surface-polymerized by hydrophilic POEM *via* ATRP. First, surface -OH groups of the ZrO_2_ are reacted with CPC at 0 °C for 24 h. The -OH groups of ZrO_2_ are substituted by -Cl groups, which are subsequently used as ATRP initiators [30,31]. The as-obtained ZrO_2_-Cl are further polymerized by hydrophilic POEM at 90 °C for 24 h *via* ATRP. The surface POEM-polymerized ZrO_2_ (ZrO_2_-POEM) are used as a template for obtaining ZrO_2_-C through carbonization at 500 °C for 30 min in Ar flow.

**Scheme 2:**
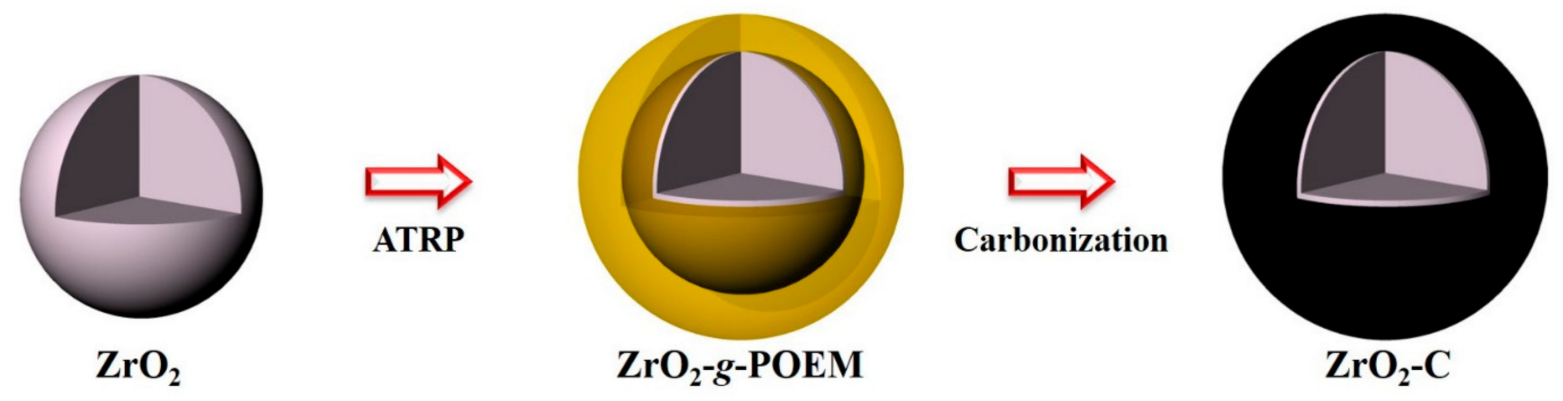
The reaction scheme for the formation of ZrO_2_-C from the pristine ZrO_2_.

Scheme 2 depicts the overall reaction scheme for obtaining ZrO_2_-C from the ZrO_2_ NPs. Photographs of the ZrO_2_, ZrO_2_-*g*-POEM, and ZrO_2_-C were shown in Figure S1. The white color of the ZrO_2_ was attributed to the large band gap of 5–5.4 eV. Upon surface polymerization with POEM, the color changed to light brown because of the surface POEM chains. After carbonization, black-colored ZrO_2_-C was clearly seen because of the surface graphitic carbon shells.

**Figure 1:**
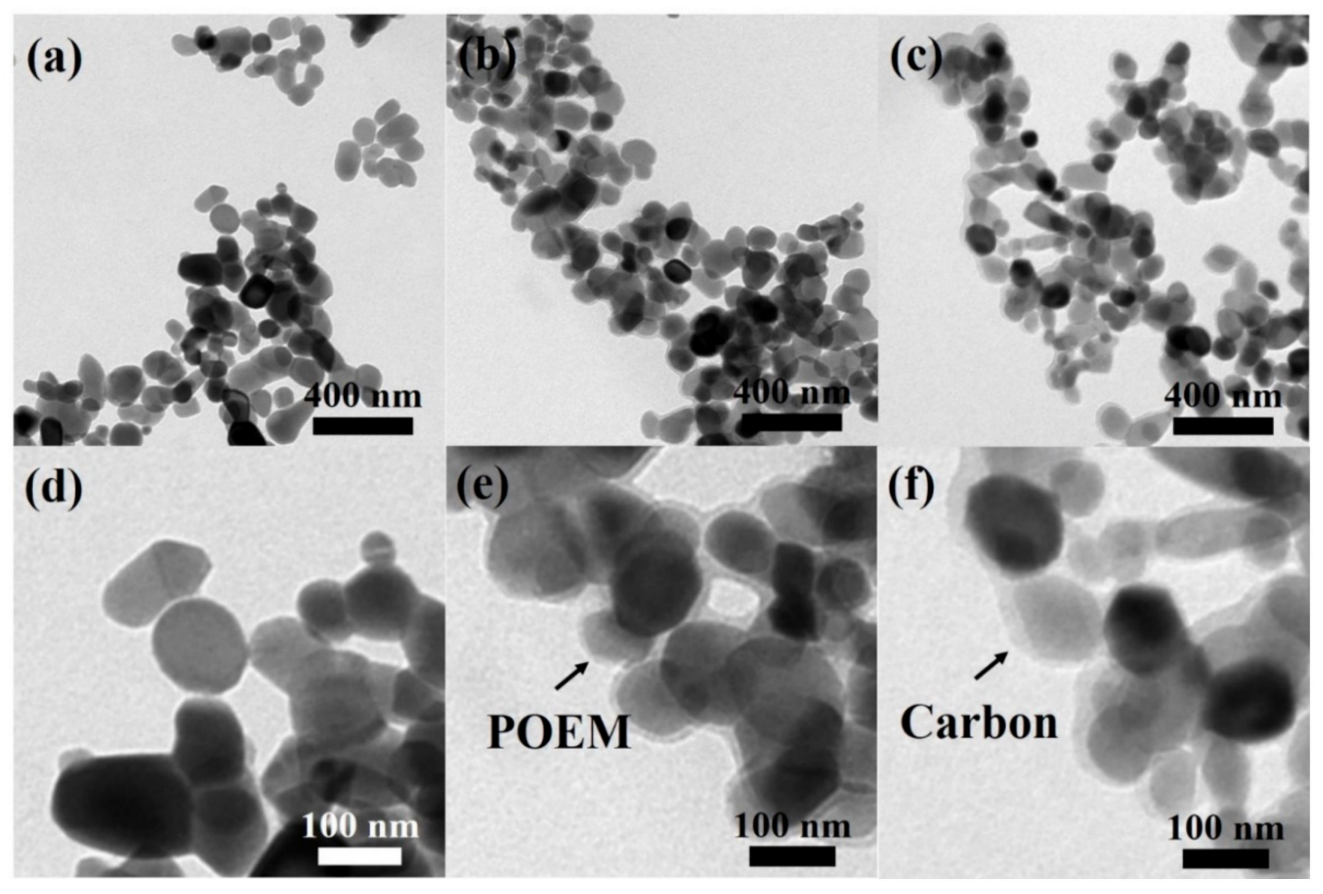
TEM image of (**a**) ZrO_2_, (**b**) ZrO_2_-*g*-POEM, (**c**) ZrO_2_-C, (**d**) magnified ZrO_2_, (**e**) magnified ZrO_2_-*g*-POEM, and (**f**) magnified ZrO_2_-C.

Figure 1 shows the TEM images of ZrO_2_, ZrO_2_-*g*-POEM, and ZrO_2_-C taken after dispersion in ethanol. The ZrO_2_ have the average diameter of 40 nm and polygonal shapes with smooth surfaces. The magnified TEM image clearly shows no surface residues on the ZrO_2_ (Figure 1d). However, after the surface-initiated ATRP reaction with POEM, ~5-nm-thick shells of POEM are visible around the surfaces of the ZrO_2_, as shown in Figure 1b. Figure 1e depicts these shells more clearly, demonstrating that the ZrO_2_ are thoroughly surface-polymerized with POEM chains. After thermal carbonization, the thin shells remain as graphitic carbon around the ZrO_2_, as shown in Figure 1c,f. Therefore, this reveals that with the introduction of the POEM polymer shells on the surfaces of the ZrO using the ATRP and following the carbonization process, the ZrO_2_-C was successfully generated.

**Figure 2:**
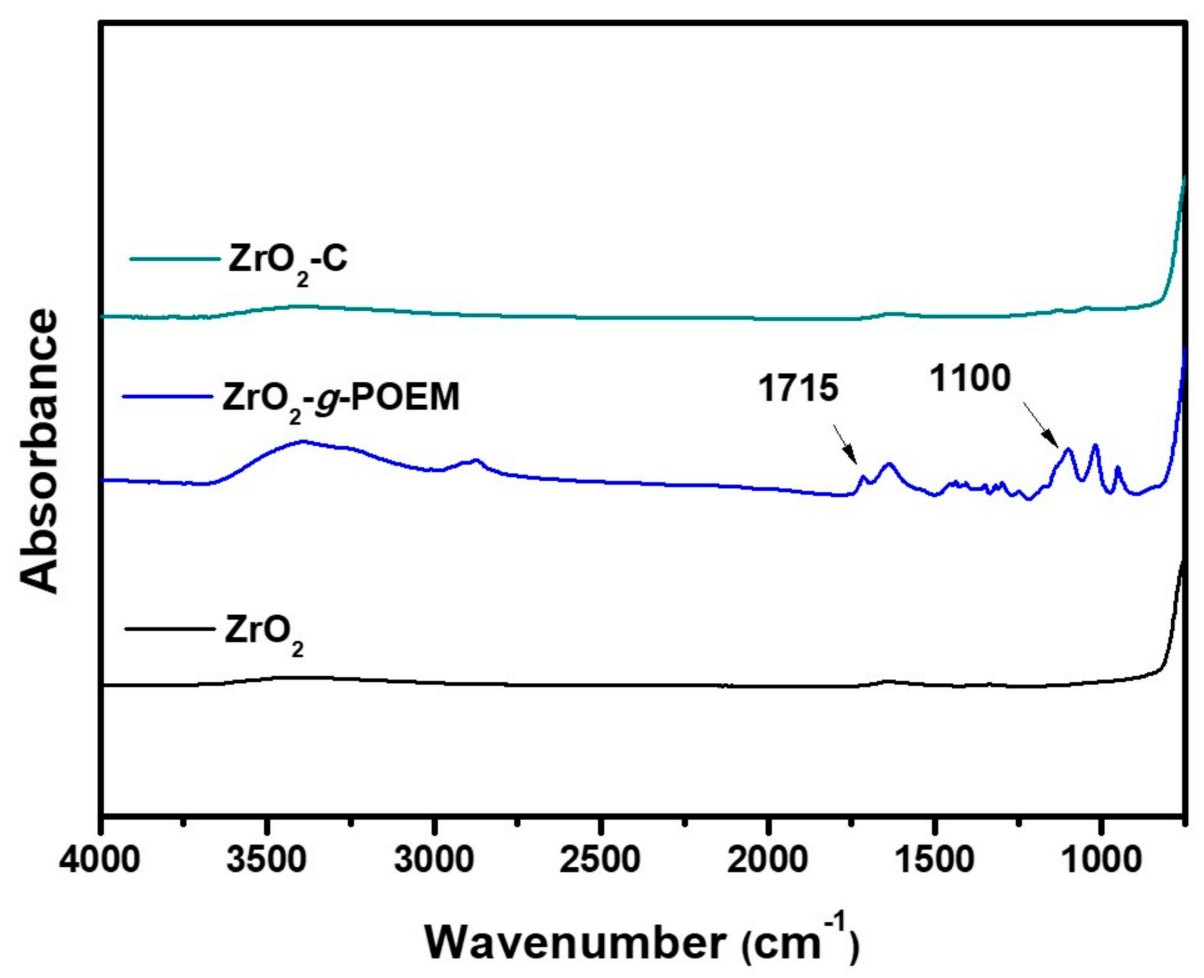
FT-IR spectra of ZrO_2_, ZrO_2_-*g*-POEM and ZrO_2_-C.

To characterize the organic groups of the various modified ZrO_2_, the FT-IR spectra of ZrO_2_, ZrO_2_-*g*-POEM, and ZrO_2_-C are shown in Figure 2. In all samples, peaks near 750 cm^−1^ are observable and attributed to the stretching vibration of the Zr-O group [32]. In the ZrO_2_-*g*-POEM, a broad 3300 cm^−1^ peak is apparent from the bending vibrations of H–O–H molecules adsorbed on the surface hydrophilic POEM chains. Furthermore, peaks at 1100 and 1715 cm^−1^ are attributed to the stretching vibrations of the ether group (C–O–C) and the carbonyl group (C=O) of surface POEM, respectively [33]. The spectrum of the ZrO_2_-C shows none of the above-mentioned peaks, demonstrating that all of the surface POEM chains are carbonized.

The weight portions of inorganic ZrO_2_ and organic POEM in ZrO_2_-*g*-POEM were easily calculated using TGA plots, as shown in Figure S2a. TGA was performed in air at 10 °C min^−1^. In the temperature range 20–200 °C, the weight fell drastically, attributed to the evaporation of water molecules adsorbed on the surfaces of the ZrO_2_-*g*-POEM. At temperatures above 250 °C, the surface POEM chains were decomposed into CO_2_ and H_2_O. Finally, almost all ZrO_2_-*g*-POEM were converted to ZrO_2_-C at the temperature of 350 °C, suggesting that the ZrO_2_-POEM included about 8 wt% POEM. To calculate the weight portion of the carbon shells on the ZrO_2_-C, the same analysis was performed in Ar as shown in Figure S2b. By similar calculations as those in Figure S2a, about 6 wt% POEM was decomposed during the carbonization process, meaning that 2 wt% of carbon remained as shells on the surface of the ZrO_2_-C. This implies that ZrO_2_-C were successfully prepared from the carbonization of ZrO_2_-POEM synthesized *via* ATRP.

**Scheme 3:**
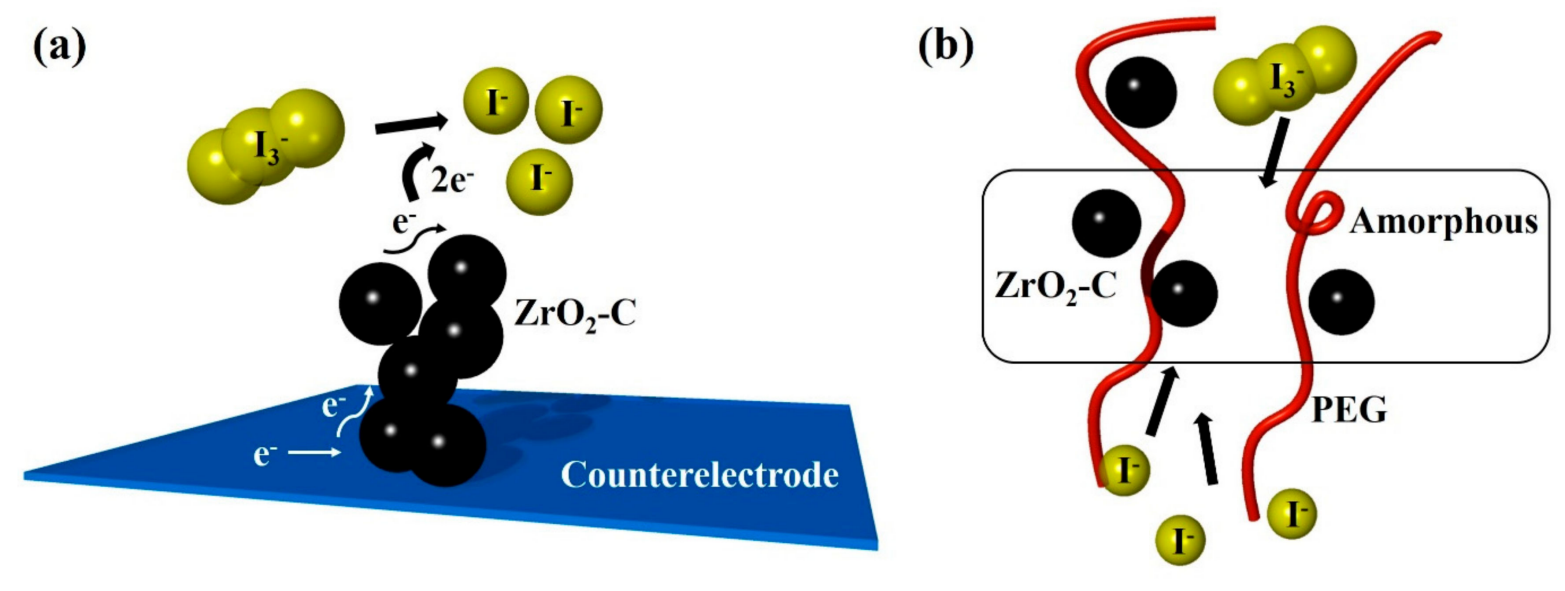
The possible mechanism of ZrO_2_-C/PGEs in (**a**) counterelectode, and (**b**) electrolyte.

Scheme 3 illustrates the possible mechanism of ZrO_2_-C/PGEs in counterelectrode and electrolyte. The carbon shells of the ZrO_2_-C in the PGEs catalyze the reduction reaction of I_3_^−^ to I^−^. Near the counter electrode, the carbon shells of the ZrO_2_-C and the counter electrode are in contact, forming a continuous electrically conductive medium, rather than an ion-conducting network accompanying the redox reactions, as shown in Scheme 3a. Ideally, zero distance is achieved between the photoanode and counterelectrode, decreasing the interfacial resistances [17,34]. Furthermore, the partial Lewis acid-base interactions with the PGEs reduce the crystallinity of the polymer and facilitate ion diffusion through the amorphous region of the electrolyte, thus enhancing ion conductivity, as shown in Scheme 3b. The bifunctional properties of ZrO_2_-C contribute to enhancing the overall conversion efficiency of DSSCs.

**Figure 3:**
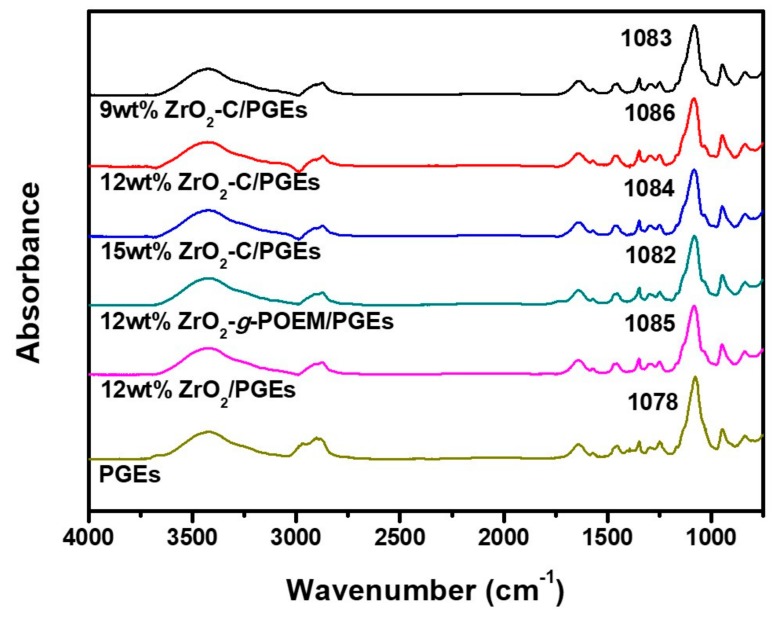
FT-IR spectra of PGEs, 12 wt% ZrO_2_/PGEs, 12 wt% ZrO_2_-*g*-POEM/PGEs, 15 wt% ZrO_2_-C/PGEs, 12 wt% ZrO_2_-C/PGEs, and 9 wt% ZrO_2_-C/PGEs.

To investigate the coordinative interactions in ZrO_2_-C/PGEs, the FT-IR spectra were measured and presented in Figure 3. Before measurement, all electrolytes were dried in vacuum oven overnight at room temperature to evaporate solvent completely. Depending on the concentrations of the ZrO_2_, ZrO_2_-*g*-POEM, and ZrO_2_-C in the PGEs, significant deviations in peaks between 1050 and 1100 cm^−1^ were observed. These arise from the interaction between Lewis basic ether groups (C-O-C) in PEGs and the Lewis acidic surfaces of the ZrO_2_. The ether peak at 1078 cm^−1^ in PGEs is shifted to 1083, 1086, and 1084 cm^−1^ after the incorporation of 9, 12, and 15 wt% ZrO_2_-C, respectively. The positive shifting of the ether peak implies increasing interactions and amorphous regions. From this perspective, the optimum concentration of ZrO_2_-C is 12 wt%. A higher concentration of ZrO_2_-C induces particle aggregation, which decreases the active sites. Interestingly, the C-O-C peak of 12 wt% ZrO_2_-*g*-POEM/PGEs is located at 1082 cm^−1^, which is lower than that of the 12 wt% ZrO_2_/PGEs (1085 cm^−1^) and ZrO_2_-C/PGEs. These FT-IR results can be explained by the fact the Lewis basic surface of POEM chains in the ZrO_2_-*g*-POEM can deactivate the Lewis acidity.

**Figure 4:**
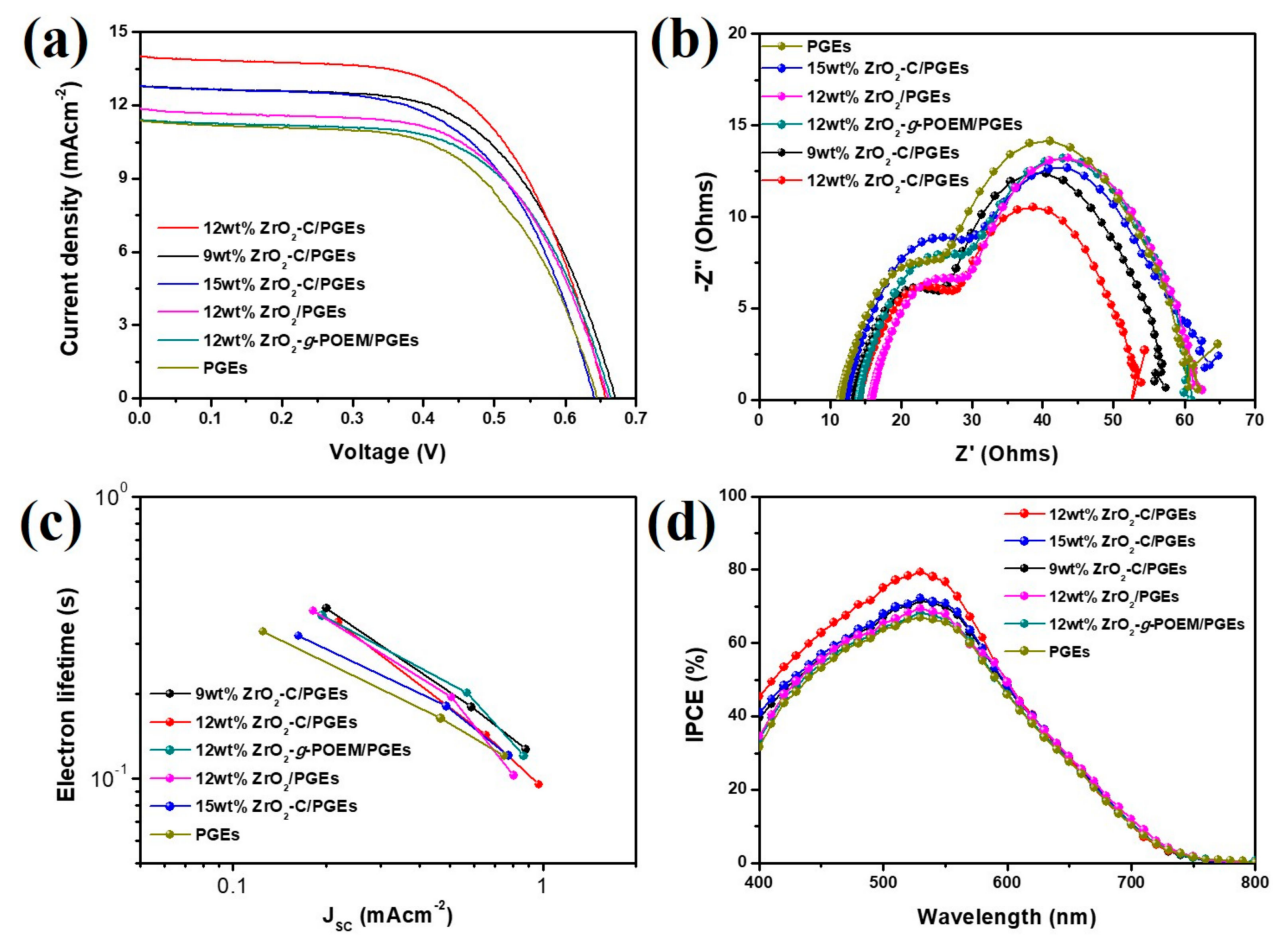
(**a**) *J-V* curves, (**b**) EIS Nyquist plots, (**c**) IMVS, and (**d**) IPCE of DSSCs fabricated with ZrO_2_-C/PGEs that were obtained under one sun illumination (AM 1.5, 100 mW cm^−2^).

To confirm the photovoltaic performances of the DSSCs utilizing ZrO_2_-*g*-POEM/PGEs, *J-V* curves under 1 sum illumination (100 mW cm^−2^) are characterized in Figure 4a. Moreover, the photovoltaic parameters of *J*_sc_, *V*_oc_, *FF*, and *η* are summarized in Table 1. The *J*_sc_ is theoretically calculated by the following equation:

*J*_sc_ = *qη*_lh_*η*_inj_*η*_col_*I*_0_ (3)

where *q* is the elementary charge, *η*_lh_ is the light-harvesting efficiency, *η*_inj_ is the electron injection efficiency, *η*_col_ is the electron collection efficiency, and *I*_0_ is the light flux. Moreover, the *V*_oc_ is estimated by the following equation:

*V*_oc_ = |*V*_FB_ − *V*_red_| (4)

where *V*_FB_ is the potential of the Fermi level of the photoanode and *V*_red_ is the standard reduction potential of a redox couple. For the 9, 12, and 15 wt% ZrO_2_-C/PEGs-based DSSCs, the *η* values reach 5.2, 5.6, and 4.9%, respectively, while that of the PGE-based DSSC is 4.4%, demonstrating that the introduction of ZrO_2_-C to the PGEs effectively improves the photovoltaic performance of DSSCs. The significantly enhanced *J*_sc_ and *V*_oc_ also contribute to the high photovoltaic performances, although *FF* remains unchanged. Two main factors cause these performance enhancements: (1) the Lewis acid-base interaction between poly (ethylene glycol) and ZrO_2_-C in the PGEs decreased the crystallinity and increased the amorphous region where redox couples could diffuse easily, yielding high ion conductivity; (2) the catalytic effect of the ZrO_2_-C enabled electron transfer to the counter electrode, thus increasing the charge-transfer rate and decreasing the interfacial resistance.

DSSCs with 12 wt% ZrO_2_-*g*-POEM and ZrO_2_/PGEs show *η* values of 4.7% and 4.8%, respectively. Surface polymerization with POEM chains deactivates the surface properties of the ZrO_2_ NPs as NFs, yielding an inferior *J*_sc_ compared to that of the 12 wt% ZrO_2_-C/PEGs and ZrO_2_/PGEs. Furthermore, the accumulation of Lewis acids such as Li^+^ salts in PGEs on the photoanode can shift the conduction band edge of TiO_2_ downward [35,36,37]. On the basis of the above studies, it can reasonably be concluded that Li^+^ salts preferentially adsorbed on the surfaces of the ZrO_2_-C instead of on the TiO_2_ photoanode is the most dominant factor in determining the enhanced *V*_oc_ (0.67 V) in 12 wt% ZrO_2_-*g*-POEM/PGE-based DSSC. The *FF* is related to not only the deep penetration of the electrolyte into the photoanode but also dark current at the photoanode, the efficiency of the recovery of the redox mediator at the counterelectrode [38,39]. Therefore, the relatively similar values of *FF* indicate that the incorporation of the ZrO_2_-*g*-POEM and ZrO_2_-C to PGEs does not relate to achieving deep penetration of the electrolyte into the photoanode. The electrochemical properties of the DSSCs are further characterized using EIS data, as shown in Figure 4b. The EIS Nyquist plots are measured from high (1 MHz) to low (0.01 MHz) frequency at the *V*_oc_ under one sun illumination (100 mW cm^−2^). The equivalent circuit model (Figure S3) includes the elements *R_S_*, *R-CE*, *R-TiO_2_*, and *W_S_*, representing the ohmic resistance of the FTO substrate (*R_S_*), interfacial charge-transfer resistance between the counter electrode and electrolyte (*R-CE*), interfacial charge-transfer resistance between the photoanode and electrolyte (*R-TiO_2_*), and the redox couple mass-transfer resistance (*W_S_*, Warburg diffusion resistance) [40,41]. The electro-kinetic properties of the DSSCs are summarized in Table 1. The DSSCs with 12 wt% ZrO_2_-*g*-POEM/PGEs, ZrO_2_/PGEs, and PGEs have similar *R-CE* values of 14.5, 13.6, and 14.2 Ω, respectively. Moreover, the DSSC with 12 wt% ZrO_2_-C/PGEs has the lowest *R-CE*, *R-TiO_2_*, and *W_S_*. This result indicates the enhanced electro-kinetic performance of the 12 wt% ZrO_2_-C/PGE-based DSSCs. While the 15 wt% ZrO_2_-C/PGE-based DSSCs has the highest *R-CE* of 16.4 Ω due to the aggregation induced by high ZrO_2_-C concentration. The mechanism underlying the photovoltaic performance improvement in the 12 wt% ZrO_2_-C/PGE-based DSSCs was investigated by measuring the electron transport and charge recombination properties through and IMVS measurements. Figure 4c shows that the τ_r_ values of the 12 wt% ZrO_2_-C/PGE-based DSSCs are always greater than those of the PEGs over the light intensity ranges. This significant improvement of electron transport and reduced charge recombination in the 12 wt% ZrO_2_-C/PGE-based DSSCs. We also confirm that the high recollection efficiency at the counterelectrode as well as slower recombination at the photoanode site of the 12 wt% ZrO_2_-C/PGE-based DSSCs which again agrees with the previous discussed EIS analysis. IPCE curves were obtained to characterize the electrochemical behavior of the DSSCs based on ZrO_2_-C/PGEs, as shown in Figure 4d. The IPCE spectra indicate that the DSSCs with the ZrO_2_-C/PGEs showed a considerably higher IPCE value than the DSSCs without the PGEs over the entire spectrum. The maximum IPCE value of the 12 wt% ZrO_2_-C/PGE-based DSSCs were 80% at λ = 520 nm, which increased up to 60% with the introduction of the PGEs. The enhanced IPCE value of the 12 wt% ZrO_2_-C/PGE-based DSSCs are attributed to the improved electron collection and injection values.

## 4. Conclusions

We demonstrated that the introduction of ZrO_2_-C in PGEs enhanced the power conversion efficiency of DSSCs. Hydrophilic POEM containing ligand oxygens were polymerized from the surface of ZrO_2_ after the introduction of the chlorine atoms *via* surface-initiated ATRP method. The ZrO_2_-*g*-POEM is used as a template to obtain the ZrO_2_-C through carbonization. The power conversion efficiency of the 12 wt% ZrO_2_-C/PGE-based DSSCs reached 5.6% at 100 mW cm^−2^; this is higher than those of PEGs (4.4%), ZrO_2_/PGEs (4.8%), and 12 wt% ZrO_2_-*g*-POEM/PGEs (4.7%). The higher cell performance of 12 wt% ZrO_2_-C/PGE-based DSSCs is due to reduced crystallinity, enhanced ion conductivity of electrolytes, decreased counterelectrode/electrolyte interfacial resistance and improved charge-transfer rate. This work highlights the potential of applying surface carbon shell-functionalized nanomaterials for the development of efficient photovoltaic devices with improved efficiency and offers future opportunities for the development of energy conversion and storage devices.

## Figures and Tables

**Table 1:** Photovoltaic and electrochemical parameters of DSSCs fabricated with ZrO_2_-C/PGEs that were obtained under one sun illumination (AM 1.5, 100 mW cm^−2^). ^a.^

Electrolyte	*V*_oc_ (V)	*J*_sc_ (mA/cm^2^)	*FF*	*η* (%)	*R_S_*	*R-CE*	*R-TiO_2_*	*W_S_*
9 wt% ZrO_2_-C/PGEs	0.67	12.8	0.61	5.2	13.1	12.8	30.4	1.4
12 wt% ZrO_2_-C/PGEs	0.66	14.0	0.61	5.6	14.1	12.6	26	1.2
15 wt% ZrO_2_-C/PGEs	0.64	12.8	0.60	4.9	12.2	16.4	32.6	3.0
12 wt% ZrO_2_-*g*-POEM/PGEs	0.67	11.5	0.61	4.7	14.0	14.5	30.6	1.7
12 wt% ZrO_2_/PGEs	0.66	11.9	0.61	4.8	15.8	13.6	31	2
PGEs	0.64	11.4	0.60	4.4	11.4	14.2	34.5	2.4

^a^ A typical cell had an active area of ca. 0.40 cm^−2^ and was masked using an aperture of the same area during the *J-V* measurements. In addition, thickness of the photoanode was approximately 10 µm.

## Supplementary Materials

The following are available online at https://www.mdpi.com/2079-4991/9/10/1418/s1, Figure S1: Photograph of ZrO_2_, ZrO_2_-*g*-POEM and ZrO_2_-C., Figure S2: TGA curves of ZrO_2_-C under (a) air and (b) Ar conditions., Figure S3: Equivalent circuit of DSSCs. Table S1: Comparison of photovoltaic parameters of DSSCs fabricated with polymer gel electrolytes reported in the literature.
