# Supplementary material for: Surface Carbon Shell-Functionalized ZrO2 as Nanofiller in Polymer Gel Electrolyte-Based Dye-Sensitized Solar Cells
Source: Nanomaterials (Basel). 2019 Oct 4;9(10):1418. doi: 10.3390/nano9101418 (PMC6836208; doi:10.3390/nano9101418)
Supplement: Supplementary file 1 [file nanomaterials-09-01418-s001.pdf]

Electronic Supplementary Information

Surface Carbon Shell-Functionalized ZrO_2 as Nanofiller in Polymer Gel Electrolyte-Based Dye-Sensitized Solar Cells

Seung Man Lim [1], Juyoung Moon [1], Gyo Hun Choi [1], Uoon Chul Baek [1], Jeong Min Lim [1], Jung Tae Park [1,*] and Jong Hak Kim [2,*]

[1] Department of Chemical Engineering, Konkuk University, 120 Neungdong-ro, Gwangjin-gu, Seoul 05029, Republic of Korea
[2] Department of Chemical and Biomolecular Engineering, Yonsei University, 50 Yonsei-ro, Seodaemun-gu, Seoul 03722, Republic of Korea
* Correspondence: jtpark25@konkuk.ac.kr (J.T.P.); jonghak@yonsei.ac.kr (J.H.K.); Tel: +82-2-450-3538, +82-2-2123-5757; Fax: +82-2-450-3504, +82-2-312-6401

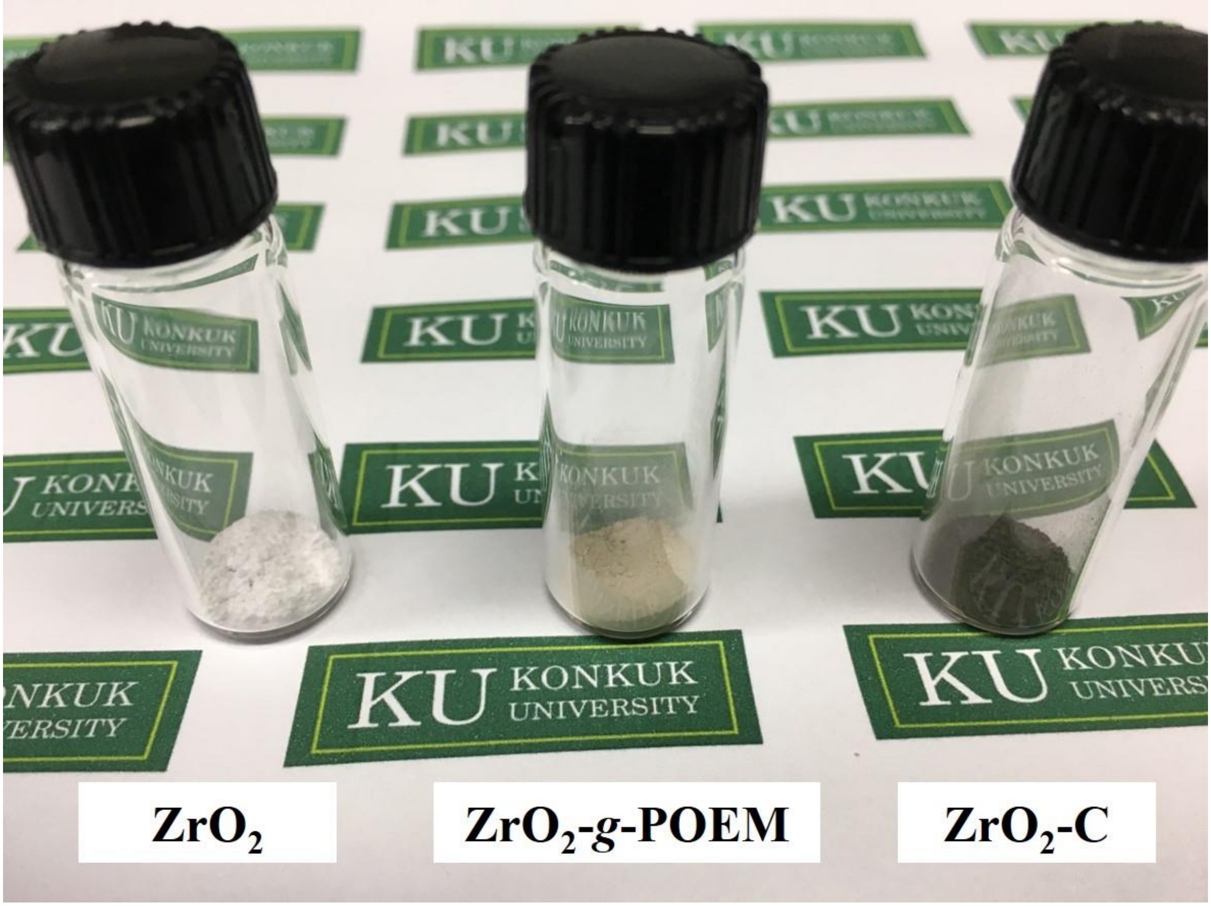

Figure 1. Photograph of ZrO_2, ZrO_2-*g*-POEM and ZrO_2-C.

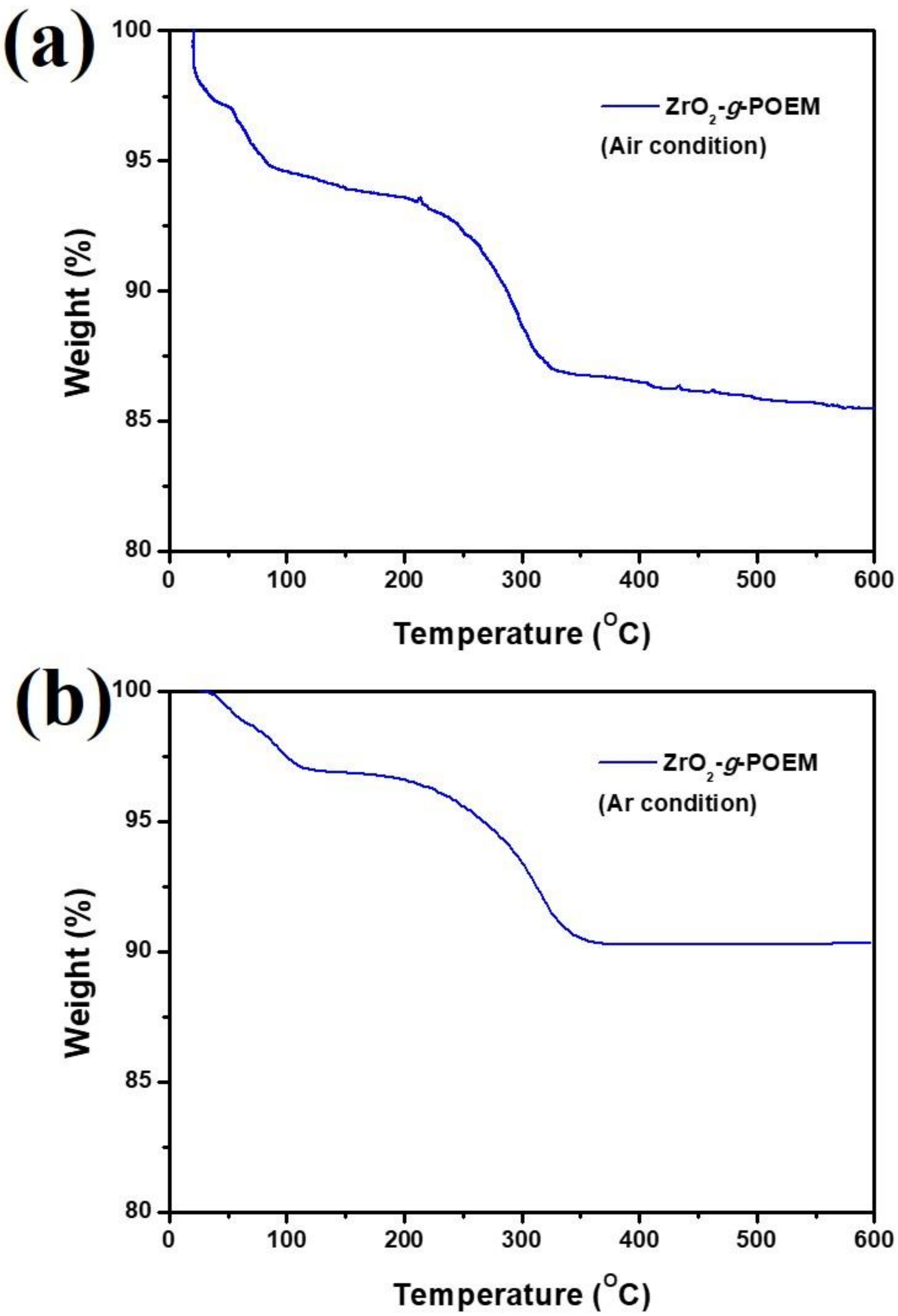

Figure 2. TGA curves of ZrO_2-C under (a) air and (b) Ar conditions.

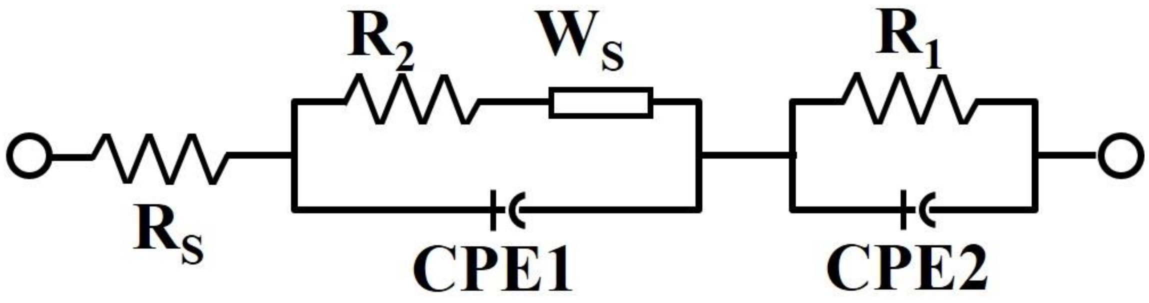

Figure 3. Equivalent circuit of DSSCs.
